# Preclinical Evaluation of NHS-Activated Gold Nanoparticles Functionalized with Bombesin or Neurotensin-Like Peptides for Targeting Colon and Prostate Tumours

**DOI:** 10.3390/molecules25153363

**Published:** 2020-07-24

**Authors:** Livia Elena Chilug, Dana Niculae, Radu Anton Leonte, Alexandrina Nan, Rodica Turcu, Cosmin Mustaciosu, Radu Marian Serban, Vasile Lavric, Gina Manda

**Affiliations:** 1 Radiopharmaceutical Research Centre, Horia Hulubei National Institute for R&D in Physics and Nuclear Engineering, 30 Reactorului Street, Magurele, 077125 Ilfov, Romania; livia.chilug@nipne.ro (L.E.C.); radu.leonte@nipne.ro (R.A.L.); cosmin@nipne.ro (C.M.); radu.serban@nipne.ro (R.M.S.); 2 Faculty of Applied Chemistry and Materials Science, University Politehnica of Bucharest, 1–7 Polizu Street, 011061 Bucharest, Romania; lavric.vasile@gmail.com; 3 National Institute for Research and Development of Isotopic and Molecular Technologies, 67–103 Donat Street, 400293 Cluj-Napoca, Romania; alex77nan@gmail.com (A.N.); rodica.turcu14@gmail.com (R.T.); 4 Faculty of Biology, University of Bucharest, 91–95 Splaiul Independentei Street, 050095 Bucharest, Romania; 5 Victor Babes National Institute of Pathology, 99–101 Splaiul Independentei Street, 050096 Bucharest, Romania; gina.manda@gmail.com

**Keywords:** gold nanoparticles, bombesin, neurotensin, PET imaging, ^68^Ga, drug delivery, prostate cancer, colon cancer

## Abstract

Recent advances and large-scale use of hybrid imaging modalities like PET-CT have led to the necessity of improving nano-drug carriers that can facilitate both functional and metabolic screening in nuclear medicine applications. In this study, we focused on the evaluation of four potential imaging nanoparticle structures labelled with the ^68^Ga positron emitter. For this purpose, we functionalized NHS-activated PEG-gold nanoparticles with ^68^Ga-DOTA-Neuromedin B, ^68^Ga-DOTA-PEG(4)-BBN(7-14), ^68^Ga-DOTA-NT and ^68^Ga-DOTA-Neuromedin N. In vitro binding kinetics and specific binding to human HT-29 colon carcinoma cells and DU-145 prostate carcinoma cells respectively were assessed, over 75% retention being obtained in the case of ^68^Ga-DOTA-PEG(4)-BBN(7-14)-AuNP in prostate tumour cells and over 50% in colon carcinoma cells. Biodistribution in NU/J mice highlighted a three-fold uptake increase in tumours at 30 min post-injection of ^68^Ga-DOTA-NT-AuNP and ^68^Ga-DOTA-PEG(4)-BBN(7-14)-AuNP compared to ^68^Ga-DOTA-NT and ^68^Ga-DOTA-PEG(4)-BBN(7-14) respectively, therewith fast distribution in prostate and colon tumours and minimum accumulation in non-targeted tissues.

## 1. Introduction

The fast development in recent years of diagnostic imaging techniques is traceable, on the one hand, to research seeking new tracers aimed for hybrid or multimodal imaging. This approach implies the integration of a structural imaging modality, such as computed tomography (CT) or magnetic resonance imaging (MRI), with a functional imaging modality, like positron emission tomography (PET) or single-photon emission computed tomography (SPECT). Among hybrid imaging combinations PET-CT is commonly preferred, as PET provides highly sensitive and quantitative information about tissue metabolic activity, while CT scanners highlight detailed and precise anatomical features. Over the last decade, special attention has been given to the development of nano-sized drug and contrast agent carriers, based on multifunctional nanoparticle (NP) systems that, due to their physicochemical properties and the large contact surface available for biomolecule functionalization, can easily improve the tumour targeting due to the enhanced permeability (EPR) effect of blood vessels that surround the tumour tissue and concentrate the amount of tracer at the tumour site [1]. These findings motivated the use of such NP systems to deliver already established radioisotopes for PET imaging, as recently reported: superparamagnetic iron oxide nanoparticles-based tracers: ^18^F-CLIO [1], ^64^Cu-DOTA-mSPIO [2,3], gold nanoparticles (AuNPs) functionalized with ^64^Cu-NODAGA [4], nitrone modified gold nanoparticles for ^18^F labelling [5], ^89^Zr-cRGDY-PEG-functionalized silica nanoparticles [6] or ^68^Ga-DOTA-TOC-AuNP [7]. A general problem encountered in the development of nano-drug structures for in vivo applications is the formation of a protein corona by non-specific absorption of opsonin proteins which facilitates the accumulation in reticuloendothelial system tissues (spleen, bone marrow, lungs, Kupffer cells of liver), instability of radiolabelled molecules or even determine the lack of targeting efficiency [8,9,10]. To overcome these problems, modification of NPs characteristics is necessary, such as size and surface chemistry but also suitable NPs’ coating with biocompatible structures, like polyethylene glycol (PEG), that improves the pharmacokinetic behaviour, changes plasma half-life of the tracer and tissue distribution. Sometimes, these difficulties can be avoided by choosing structures that meet all the above requirements, but it is still not enough and the optimization of the radiolabelled vector becomes necessary (radioisotope-labelled biological active molecule) as well as a full characterization of the targeting properties of the chosen biomolecule to the expected target tissue.

Among various radioisotopes used in nuclear medicine imaging, ^68^Ga has the characteristics of positron emission and low photon emission, which enable high detection resolution and sensitivity (T_1/2_ = 67.83 min, E_β+_ = 1.899 MeV). ^68^Ga can be obtained either by irradiation of ^68^Zn targets at cyclotrons [11], or, more often, by elution of a ^68^Ge/^68^Ga radionuclide generator [7]. Gallium can form stable complexes with a large number of ligands. Coupling of ^68^Ga to biological vectors demands proper chelating agents, capable of stably binding the radioisotope to the biologically active molecule. For the studies underlying this work, 1,4,7,10-tetraazacyclododecane-1,4,7,10-tetraacetic acid (DOTA) was used as the chelator for ^68^Ga.

The current study focuses on preclinical in vitro and ex vivo evaluation on colon and prostate carcinoma cells of four different peptides labelled with ^68^Ga, functionalized on gold nanoparticles, with potential to be used in multimodal imaging application. We labelled two groups of homologous peptides: PEG(4)-bombesin (7–14) (Gln-Trp-Ala-Val-Gly-His-Leu-Met-NH_2_) and its counterpart neuromedin B (Gly-Asn-Leu-Trp-Ala-Thr-Gly-His-Phe-Met-NH_2_), also neurotensin (Pyr-Leu-Tyr-Glu-Asn-Lys-Pro-Arg-Arg-Pro-Tyr-Ile-Leu-OH) and neuromedin N (Lys-Ile-Pro-Tyr-Ile-Leu). Bombesin receptors family consists of three subtypes of receptors: the neuromedin B (NMB) receptor (BBRS1 receptor), the gastrin-releasing peptide (GRP) receptor (BBRS2 receptor) and bombesin receptor subtype 3 (BBRS3). They are frequently over-expressed and released by breast tumours, prostate tumours, ovarian cancers, and small lung cancer. The bombesin-like peptide neuromedin B (NMB) is present in the central nervous system and gastrointestinal tract of mammals, stimulates smooth muscle contraction and is an autocrine growth factor in non-small cell lung cancer [12]. Neurotensin (NT) and neurotensin-like peptide neuromedin N (NMN) are derived from the same polypeptide precursor [13], neurotensin receptors (NTRS1, NTRS2 and NTRS3) being overexpressed by lung, breast cancer, colon or prostate cancer. To demonstrate the ability of gold nanoparticles to improve the in vivo uptake of tracers, we selected the stabilised sequence 7–14 of the bombesin peptide, with improved pharmacokinetics via the oligoethylene glycol spacer PEG_4_ [14] but also its homologous peptide neuromedin B for comparison. The unmodified neurotensin peptide has previously been shown to have faster in vivo kinetics due to degradation under the action of metalloendopeptidase, more stable analogues being recently developed [15,16]. Thus, we selected a peptide with already improved pharmacokinetics (and its counterpart), but also a peptide with a shorter biological time to demonstrate that in all cases gold NPs may be able to improve the tumour-targeting. In the present study we functionalized gold nanoparticles conjugated with PEG and activated with NHS groups aimed for better stability of the binding with selected peptides. We then assessed in vitro cell specific and non-specific uptake on HT-29 colon and DU-145 prostate carcinoma cells, which express bombesin and neurotensin receptors on their surface. Ex vivo biodistribution in NU/J mice was performed to assess the targeting potential of each of the investigated nanostructures.

## 2. Results

### 2.1. Peptides Labelling

The overall process, including elution, labelling and purification procedures, took less than 25 min to the final product, on an automated synthesis module. Irrespective of peptide, the radiolabelling processes were set to similar parameters (temperature, pH, reaction time, molar ratios) and purification steps. This led to slightly different results in terms of preparation yields, as presented in Table 1.

### 2.2. Quality Control of ^68^Ga-Labelled Peptides

**Figure A1:**
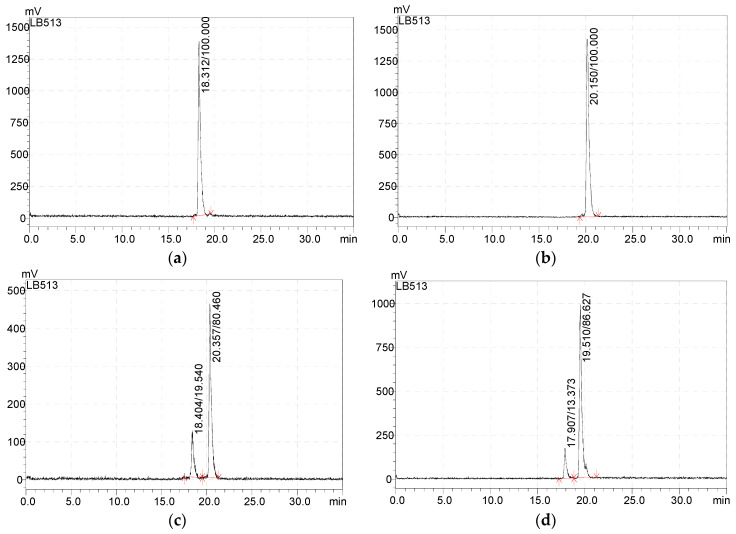
Radiochemical purity of ^68^Ga labelled peptides at EOS: (**a**) ^68^Ga-DOTA-NT; (**b**) ^68^Ga-DOTA-MN; (**c**) ^68^Ga-DOTA-PEG(4)-BBN(7-14); (**d**) ^68^Ga-DOTA-NMB.

The radiochemical purity (RCP) of the labelled biomolecules, assessed at the end of preparation and purification process, showed no unbound ^68^Ga and no degradation molecules resulted from preparation, in the final product. In case of ^68^Ga-DOTA-NT and ^68^Ga-DOTA-NMN, RCP is 100%. The radio-chromatogram profiles of ^68^Ga-DOTA-PEG(4)-BBN(7-14) and ^68^Ga-DOTA-NMB are similar to corresponding chromatograms of the cold peptides as per certificates of analysis provided by the producers, no other radiolabelled by-products being detected; in these cases, RCP was higher than 80% and 85%, respectively. Appendix A. Figure A1 presents radio-chromatograms of each studied compound at the end of synthesis (EOS).

RCP was checked every hour up to 4 h after the end of synthesis, with no degradation observed. All labelled molecules have qualified for biological studies. The pH of the final compound was neutral (pH = 7 ± 0.2), thus proper for direct injection.

### 2.3. Physicochemical Characterisation of Peptide-Functionalized Gold Nanoparticles

#### 2.3.1. Dynamic Light Scattering (DLS)

**Figure 1:**
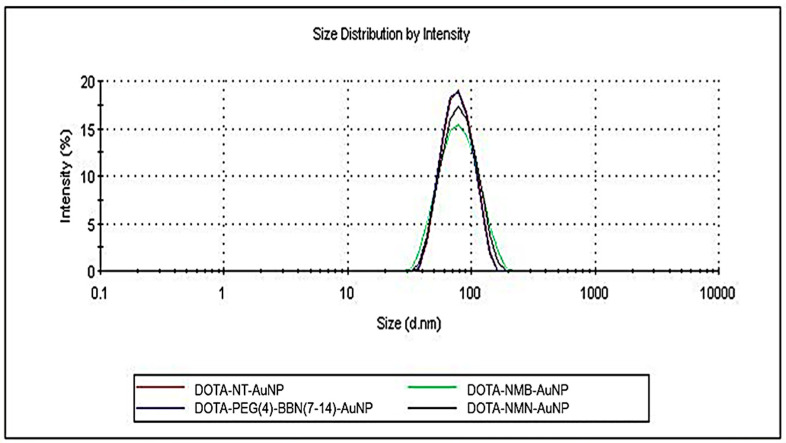
DLS spectra-size distribution by intensity of gold nanoparticles functionalized with peptides: DOTA-NT-AuNP (red), DOTA-NMN-AuNP (black), DOTA-NMB-AuNP (green) and DOTA-PEG(4)-BBN(7-14)-AuNP (blue).

The reaction between DOTA-peptides and NHS-activated PEG-AuNPs was monitored by detecting changes in nanoparticles size as a peptide layer is formed around nanoparticles. The initial hydrodynamic diameter of bare PEG-AuNPs was 42.56 nm, and after functionalization increased to approximately 73 nm (Figure 1). Details of each nanostructures physical properties are listed in Table 2. The electric potential at the boundary of the double layer (counter ions and Stern layer) of nanoparticles, also known as zeta potential is higher than −44 mV. Thus, the peptide-functionalized nanoparticles have sufficient repulsive force to anticipate a good physical stability.

#### 2.3.2. FTIR Spectra

**Figure 2:**
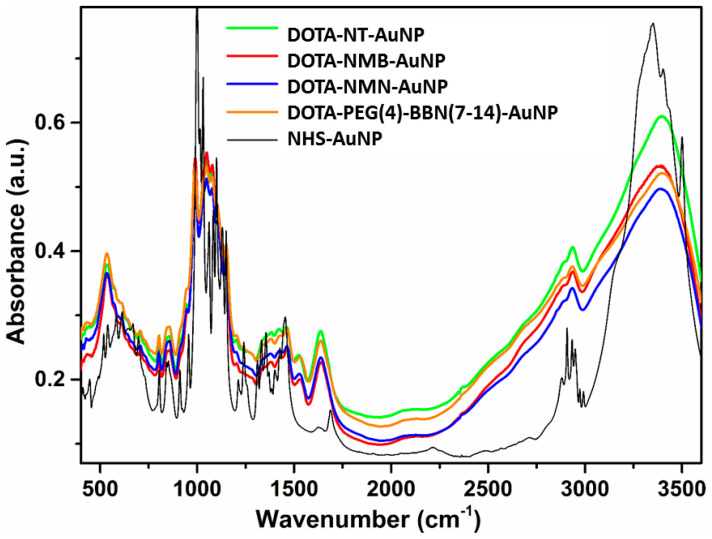
FTIR absorption spectra of DOTA-NT-AuNP (green), DOTA-NMB-AuNP (red), DOTA-NMN-AuNP (blue) and DOTA-PEG(4)-BBN(7-14)-AuNP (orange) and NHS-AuNP (black).

In Figure 2 comparison between the FTIR spectra of NHS-AuNP DOTA-NT-AuNP, DOTA-NMB-AuNP, DOTA-NMN-AuNP and DOTA-PEG(4)-BBN(7-14)-AuNP is presented. As can be seen, the signals in the FTIR spectrum of the starting NHS-AuNP nanoparticles are distinct from those present in the spectra of DOTA peptide-functionalized AuNPs, due to the fact that the FTIR spectra were not measured for quantitative analysis. The signals in the NHS-AuNPs spectrum are more intense than those present in the DOTA peptide-functionalized AuNP spectra. The FTIR spectra of the gold nanoparticles samples contain the characteristic absorption bands of both constituents, namely DOTA-peptide and AuNPs. The relatively intense absorption band from AuNPs FTIR spectrum located around 605 cm^−1^ is typical for Au nanoparticles. In the FTIR spectra of the DOTA-peptide functionalized AuNPs is shifted to lower wavenumber (538 cm^−1^), due to the attachment of the DOTA-peptide complex on the AuNPs. The characteristic bands of the peptide conjugated with the chelating agent DOTA appear in the range 600–3600 cm^−1^. The intense band at 3380 cm^−1^ is attributed to the free NH stretching band and H-bonded NH groups from the peptide. Bands at 2914 cm^−1^ and 2855 cm^−1^ region are arising from C-H and CH_2_ stretching bonds of the organic layer found at the AuNPs surface. The band at 1640 cm^−1^ in the spectra corresponds to C=O stretching, highly indicating the presence of peptides on the nanoparticles surface. The same C=O group present in NHS ester molecule provides in the FTIR spectrum of NHS-AuNP two weak absorption bands with a peak at 1623 cm^−1^ and the other at 1690 cm^−1^; this fact indicates the covalent linkage of the DOTA-peptides on AuNPs surface. The absorption band situated at 1547 cm^−1^ is ascribed to the collective vibration of C=C/C-C of peptide chains. The band at 1455 cm^−1^ was assigned for N-H/C-N stretch vibration present in the amide linkages of the proteins.

### 2.4. Cytotoxicity Evaluation

We performed a preliminary study for assessing the potential cytotoxic effects of the investigated types of NPs, both bare NPs (AuNP) and peptide-loaded NPs (DOTA-PEG(4)-BBN(7-14)-AuNP, DOTA-NMB-AuNP, DOTA-NMN-AuNP, DOTA-NT-AuNP), on normal cells (human proliferating CRL9855 monocytes) and tumour cells (human colon carcinoma HT-29 cells and human prostate carcinoma DU-145 cells). The MTS reduction test was used to evaluate the number of metabolically active cells in culture, under various experimental conditions. NPs were tested at 0.25 mg/mL and 0.50 mg/mL, that are higher than those used for the in vitro uptake and retention study (0.10–0.15 mg/mL). These concentrations are also considered high as compared to the concentration domain in which AuNPs are generally tested in vitro (0.005–0.025 mg/mL) [17].

**Figure A2:**
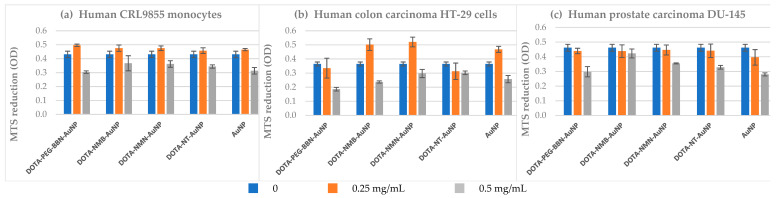
MTS reduction by human CRL955 monocytes (**a**), human colon carcinoma HT-29 cells (**b**) and human prostate DU-145 carcinoma cells (**c**) incubated for 48 h with bare NPs (AuNP) or with peptide-loaded NPs (DOTA-PEG-BBN-AuNP, DOTA-NMB-AuNP, DOTA-NMN-AuNP, DOTA-NT-AuNP). Results are presented as mean ± SEM (standard error of the mean) for triplicate samples.

At the concentration of 0.25 mg/mL, the investigated peptide-loaded NPs did not significantly change MTS reduction in normal or tumour cells, exerting almost the same effect as AuNP. In turn, higher concentrations (0.50 mg/mL) of peptide-loaded NPs and AuNP had an inhibitory action on MTS reduction (Appendix A, Figure A2). Results indicate that a concentration of 0.25 mg/mL is biocompatible with the investigated types of cells, and therefore lower concentrations might be safely used for functional studies on ^68^Ga-labelled NPs addressing cellular uptake and retention. In turn, higher NPs concentrations (0.50 mg/mL) can trigger a significant reduction in the number of metabolically active cells (Appendix A, Figure A2), indicating that accumulation of NPs in blood, after the intravenous inoculation, or in organs, might have deleterious effects. This might be instead a therapeutic benefit in tumour cells, whose viability and proliferation might be limited by the investigated NPs, even in the absence of the radioisotope.

**Figure A3:**
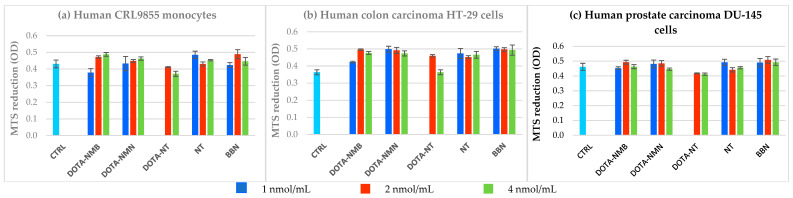
The effect exerted by neuropeptides on MTS reduction by human CRL955 monocytes (**a**), human colon carcinoma HT-29 cells (**b**) and human prostate DU-145 carcinoma cells (**c**) in 48 h cultures. Results are presented as mean ± SEM (standard error of the mean) for triplicate samples.

Considering that peptides loaded on NPs might detach in the blood flow or when diffusing into organs, we have investigated their direct impact on MTS reduction (Appendix A, Figure A3). DOTA-NMB, DOTA-NMN, NT and BBN do not alter MTS reduction by the investigated normal and tumour cells in the concentration domain of 1–4 nmol/mL, whereas DOTA-NT has only a slightly inhibitory action at concentrations lower than 2 nmol/mL.

In conclusion, the tested peptide-loaded NPs appear to be biocompatible with normal proliferating cells and tumour cells, and do not raise any cytotoxicity issues in the absence of the radioisotope.

### 2.5. In Vitro Binding Assay

#### 2.5.1. Uptake and Retention

**Figure 3:**
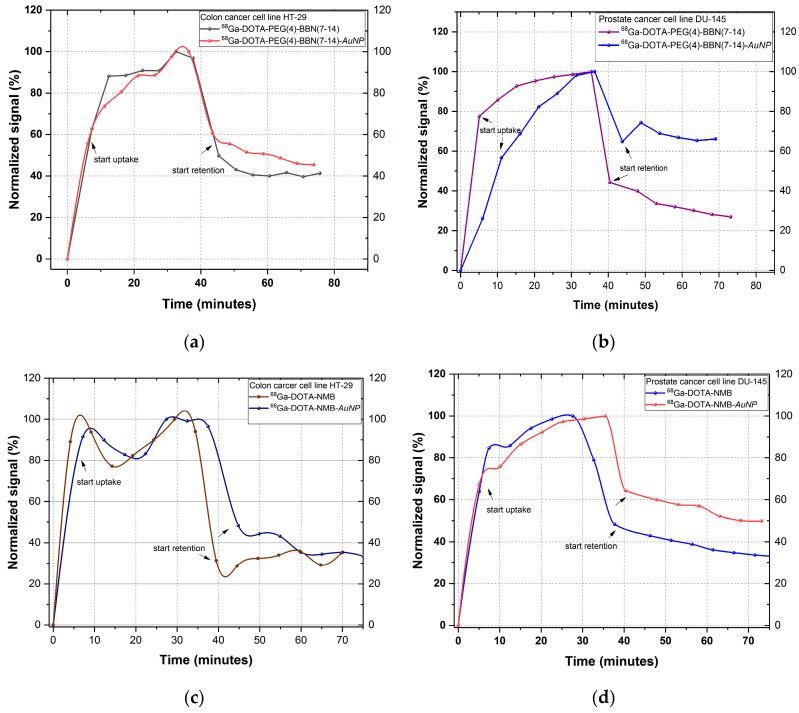

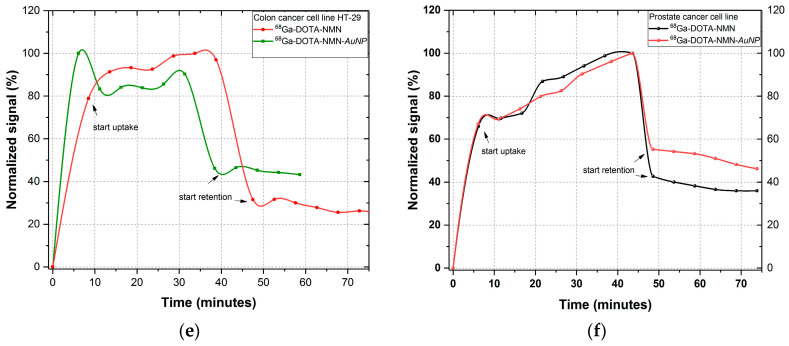
In vitro comparative uptake and retention of: (**a**) ^68^Ga-DOTA-PEG(4)-BBN(7-14) and ^68^Ga-DOTA-PEG(4)-BBN(7-14)-AuNP on HT-29 cell line; (**b**) ^68^Ga-DOTA-PEG(4)-BBN(7-14) and ^68^Ga-DOTA-PEG(4)-BBN(7-14)-AuNP on DU-145 cell line; (**c**) ^68^Ga-DOTA-NMB and ^68^Ga-DOTA-NMB-AuNP on HT-29 cell line; (**d**) ^68^Ga-DOTA-NMB and ^68^Ga-DOTA-NMB-AuNP on DU-145 cell line; (**e**) ^68^Ga-DOTA-NMN and ^68^Ga-DOTA-NMN-AuNP on HT-29 cell line; (**f**) ^68^Ga-DOTA-NMN and ^68^Ga-DOTA-NMN-AuNP on DU-145 cell line.

The binding kinetic (uptake and retention) of the ^68^Ga-labelled peptides and peptide-functionalized gold nanoparticles we investigated using rotating radioimmunoassay. 2–3 nmol of each peptide labelled with ^68^Ga was incubated on DU-145 prostate and HT-29 colon cancer cells. After uptake measurement, the radioactive medium was removed and the cells were washed with fresh DMEM-F12 medium. The cell retention is measured after this process, during each rotation of the Petri dish support the detector is measuring only the signal coming from the tracers bound to cells. The results are reported as measured signal during the time of analysis (where t = 0 min is the time of incubation), as illustrated in Figure 3. Following the analysis of each peptide, we can easily observe that the uptake of both nanostructured and peptide-based pharmaceuticals is increasing almost exponentially with the incubation time, which indicates a good dynamic on the tumour cells. A lower outcome is obtained for ^68^Ga-DOTA-NMB and ^68^Ga-DOTA-NMB-AuNP on colon cancer cell line HT-29, for which the uptake profile follows an up and down trend, an indicator of unstable binding to neuromedin B receptors (NMB, BBRS2, BBRS3).

The retention level is illustrated after the indicator arrows and reflects the percent of activity retained by the cells from the maximum counted during uptake, after hot medium removal and cells washing step. This part of analysis is a screening that enable evaluation of peptide potential to form stable bonds with its specific receptors. The retention of all peptides exceeds 30%, for most the level being maintained until the end of the analysis (approx. 75 min). The only peptide for which retention level fluctuates is NMB incubated on HT-29 cells, which indicates an unstable or weak peptide-receptor bond. From the uptake-retention trend of functionalized AuNPs, we can observe that gold nanoparticles improve cell retention of ^68^Ga-labelled peptides without affecting the ability of peptides to bind to receptors. A significant increase was obtained for ^68^Ga-DOTA-PEG(4)-BBN(7-14)-AuNP on DU-145 prostate cancer cells, that reaches 75% compared to approximately 38% for ^68^Ga-DOTA-PEG(4)-BBN(7-14).

#### 2.5.2. Specific Binding

**Figure 4:**
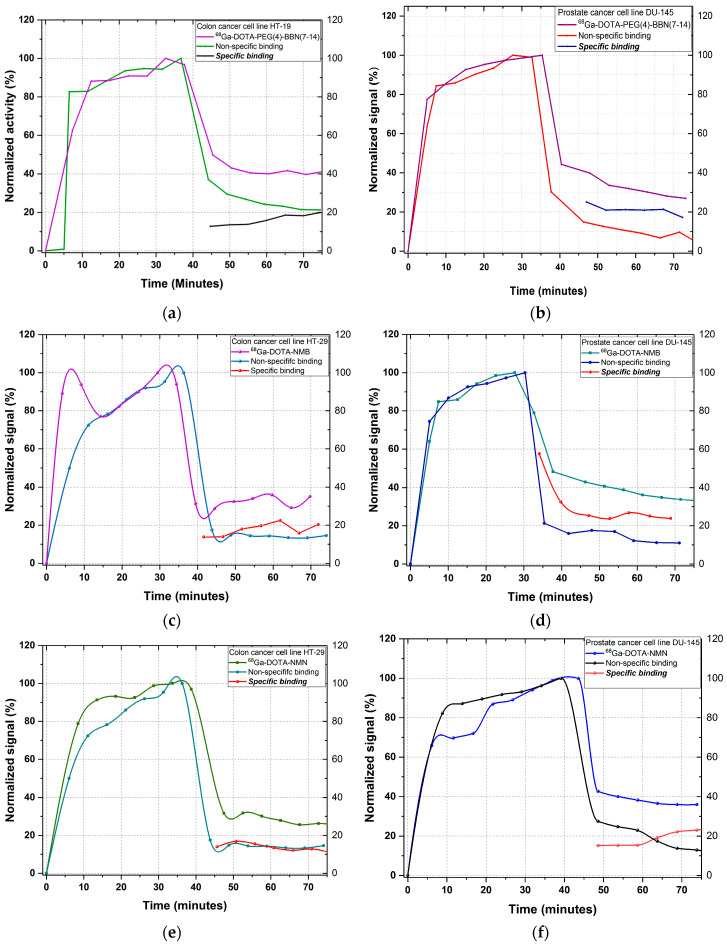
In vitro specific binding evaluation of: (**a**) ^68^Ga-DOTA-PEG(4)-BBN(7-14) on HT-29 cell line; (**b**) ^68^Ga-DOTA-PEG(4)-BBN(7-14) on DU-145 cell line; (**c**) ^68^Ga-DOTA-NMB on HT-29 cell line; (**d**) ^68^Ga-DOTA-NMB on DU-145 cell line; (**e**) ^68^Ga-DOTA-NMN on HT-29 cell line; (**f**) ^68^Ga-DOTA-NMN on DU-145 cell line.

The evaluation of specific binding of a peptide towards cell membrane receptors is strongly dependent upon the density of receptors, their conformation (active or inactive) and the concentration of inhibitor used to block the receptors prior to peptide incubation [18,19]. For specific binding determination, the receptors for each peptide were blocked with several antagonists: ML-18 and PD176252 (K_d_ = 1 nM) for bombesin BBRS1, BBRS2 and BBRS3 receptors; SR48692 (K_d_ = 15 nM), SR142948 (K_d_ = 10 nM) and NTRC 824 (K_d_ = 38 nM) for neurotensin NTRS1, NTRS2 and NTRS3 receptors. The incubation time of antagonists was 1–1.5 h, then the medium was removed and the cells were washed and supplemented with ~2 mL fresh culture medium. The non-specific uptake and retention of each ^68^Ga-labelled peptide was subsequently evaluated using the same protocol as the one used for total binding. The binding kinetics profiles for non-specific binding of each peptide and the total binding are presented in Figure 4. Specific binding is the difference between the total binding and non-specific binding. We can observe that the specific retention of the peptides is about 20%, which represents half of the total binding for each ^68^Ga-labelled peptide.

### 2.6. Ex Vivo Biodistribution

#### 2.6.1. ^68^Ga-DOTA-PEG(4)-BBN(7-14)/^68^Ga-DOTA-PEG(4)-BBN(7-14)-AuNP

**Figure 5:**
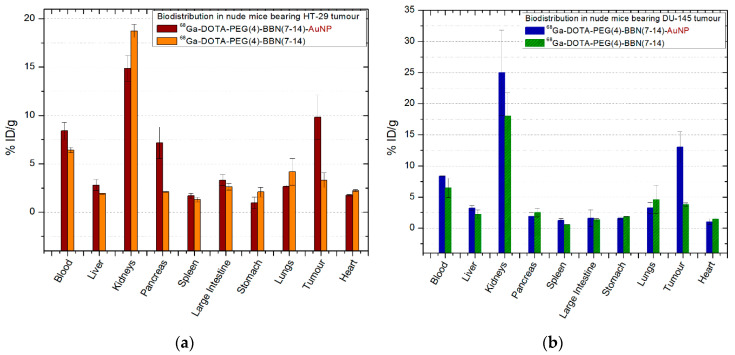
Comparative ex vivo biodistribution after 30 min post-injection of ^68^Ga-DOTA-PEG(4)-BBN(7-14)/^68^Ga-DOTA-PEG(4)-BBN(7-14)-AuNP: (**a**) in nude mice bearing colon tumour; (**b**) in nude mice bearing prostate tumour. Data are presented as mean ± SD (*n* = 4).

The comparative biodistribution of ^68^Ga-DOTA-PEG(4)-BBN(7-14)/^68^Ga-DOTA-PEG(4)- BBN(7-14)-AuNP is presented in Figure 5, all the experimental data being included in Appendix B, Table A1 and Table A2. After 30 min post-injection (p.i.) a fast clearance of ^68^Ga-DOTA-PEG(4)-BBN(7-14) was observed in the kidneys (18.74 ± 0.67% ID/g) and in the blood (6.42 ± 0.27% ID/g). Compared to that, ^68^Ga-DOTA-PEG(4)-BBN(7-14)-AuNP shows higher level in the blood (8.24 ± 0.86% ID/g) and lower in the kidneys (14.86 ± 1.32% ID/g), considered significant as *p* = 0.047. Additionally, 4.17 ± 1.37% ID/g of ^68^Ga-DOTA-PEG(4)-BBN(7-14) is found in the lungs. Instead, the activity in the pancreas is 3.37 times higher if BBN-functionalized gold nanoparticles are used instead of ^68^Ga-DOTA-PEG(4)-BBN(7-14) (*p* = 0.022). A slight increase was registered in the spleen (1.69 ± 0.26% ID/g for AuNP compared to 1.31 ± 0.22% ID/g for BBN, *p* = 0.025), liver (2.81 ± 0.57% ID/g for AuNP compared to 1.92 ± 0.07% ID/g for BBN, *p* = 0.014), and large intestine (3.31 ± 0.56% ID/g for AuNP compared to 2.63 ± 0.34 for BBN, *p* = 0.021).

A significant increase in activity (*p* = 0.007) is observed at the tumour site, where activity triples after ^68^Ga-DOTA-PEG(4)-BBN(7-14)-AuNP use, reaching 9.82 ± 2.28% ID/g from 3.31 ± 0.76% ID/g with ^68^Ga-DOTA-PEG(4)-BBN(7-14).

A similar behaviour was observed in mice inoculated with prostate tumour cells (Figure 5b), the lungs and heart having lower values after ^68^Ga-DOTA-PEG(4)-BBN(7-14)-AuNP injection compared to ^68^Ga-DOTA-PEG(4)-BBN(7-14) (*p* = 0.042), and increasing activity in the liver and large intestine. Prostate tumour retained 13.01 ± 2.49% ID/g of ^68^Ga-DOTA-PEG(4)-BBN(7-14)-AuNP, three times (>3.44) higher than ^68^Ga-DOTA-PEG(4)-BBN(7-14) uptake (3.78 ± 0.27% ID/g), the values being significant as the *p*-value is *p* = 0.013.

#### 2.6.2. ^68^Ga-DOTA-NMB/^68^Ga-DOTA-NMB-AuNP

**Figure 6:**
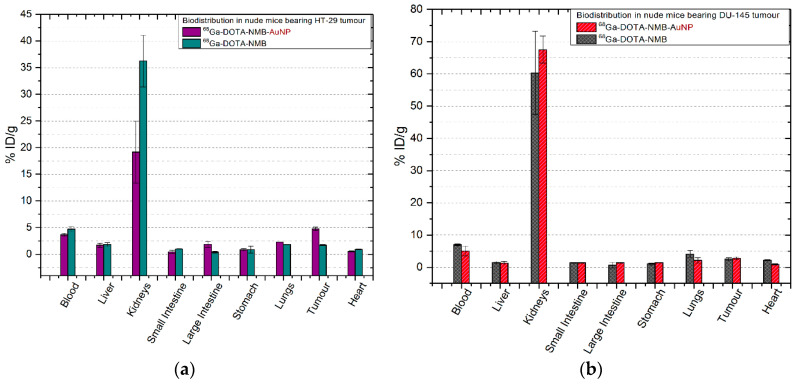
Comparative ex vivo biodistribution after 30 min post-injection of ^68^Ga-DOTA-NMB/^68^Ga-DOTA-NMB-AuNP: (**a**) in nude mice bearing colon tumour; (**b**) in nude mice bearing prostate tumour. Data are presented as mean ± SD (*n* = 4).

In both mice with colon tumours and those with prostate tumours, ^68^Ga-labelled neuromedin B peptide(-AuNP) is quickly removed from the circulatory system and after only 30 min p.i. both tracers are found in the kidneys (60.28 ± 13.01% ID/g of ^68^Ga-DOTA-NMB and 67.51 ± 4.16% ID/g for ^68^Ga-DOTA-NMB-AuNP, with *p* = 0.017) and only 7.00 ± 0.24% ID/g ^68^Ga-DOTA-NMB in the blood. This observation is also corroborated with the results obtained in the other tissues, being significantly lower compared to its counterparts ^68^Ga-DOTA-PEG(4)-BBN(7-14) and ^68^Ga-DOTA-PEG(4)-BBN(7-14)-AuNP (*p* = 0.005). The uptake of ^68^Ga-DOTA-NMB (Figure 6b) by the prostate tumour is 2.54 ± 0.42% ID/g and 2.79 ± 0.44% ID/g for ^68^Ga-DOTA-NMB-AuNP (*p* = 0.043). Similar values were recorded in colon tumours (Figure 6a), as 1.73 ± 0.08% ID/g ^68^Ga-DOTA-NMB and 4.75 ± 0.32% ID/g of ^68^Ga-DOTA-NMB-AuNP were reported (*p* = 0.026).

#### 2.6.3. ^68^Ga-DOTA-NT/^68^Ga-DOTA-NT-AuNP

**Figure 7:**
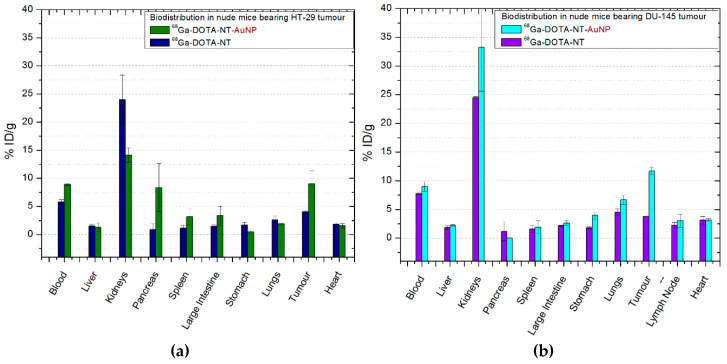
Comparative ex vivo biodistribution after 30 min post-injection of ^68^Ga-DOTA-NT/^68^Ga-DOTA-NT-AuNP: (**a**) in nude mice bearing colon tumour; (**b**) in nude mice bearing prostate tumour. Data are presented as mean ±SD (*n* = 4).

^68^Ga-DOTA-NT uptake in colon and prostate tumours (Figure 7a,b) is similar as 4.06 ± 0.27% ID/g of tracer was measured in the colon tumours of mice, and 3.78 ± 0.05% ID/g in prostate tumours. ^68^Ga-DOTA-NT-AuNP shows comparative results with ^68^Ga-DOTA-PEG(4)-BBN(7-14)-AuNP being a 3-fold increase of activity in prostate tumours (11.7 ± 0.59% ID/g) and two times higher in the colon tumours (9.04 ± 2.29% ID/g). In both mice with colon tumours and those with prostate tumours, the excretion of neurotensin is fast, after 30 min p.i. about 25% ID/g being found in the kidneys and 7–9% ID/g in the bloodstream.

Functionalized gold nanoparticles influenced the increase of the amount of tracer in the spleen (3.19 ± 1.39% ID/g in mice with colon cancer and 1.92 ± 0.11% ID/g in those with prostate tumours, *p* = 0.035) and large intestine (2.59 ± 0.44% ID/g in mice with colon tumours and 3.37 ± 1.6% ID/g in those with prostate cancer, *p* = 0.021). In addition, a significant uptake (*p* = 0.016) was measured in the pancreas to three of the mice group (*n* = 4) inoculated with HT-29 colon cancer cells. Also, in the inguinal lymph node of mice with prostate cancer there is a high percentage of ^68^Ga-DOTA-NT (2.23 ± 0.49% ID/g) as well as ^68^Ga-DOTA-NT-AuNP (3.04 ± 1.13% ID/g), although, the result is not statistically significant (*p* = 0.7).

#### 2.6.4. ^68^Ga-DOTA-NMN/^68^Ga-DOTA-NMN-AuNP

**Figure 8:**
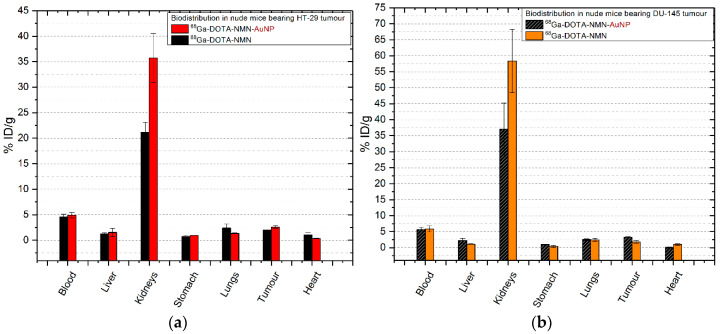
Comparative ex vivo biodistribution after 30 min post-injection of ^68^Ga-DOTA-NMN/^68^Ga-DOTA-NMN-AuNP: (**a**) in nude mice bearing colon tumour; (**b**) in nude mice bearing prostate tumour. Data are presented as mean ± SD (*n* = 4).

The results obtained for mice injected with neuromedin N and gold nanoparticles functionalized with neuromedin N are illustrated in Figure 8. The pharmacokinetic of neuromedin N peptide in mice inoculated with HT-29 colon cancer cells is predominated by the fast clearance of tracers from the bloodstream (4.59 ± 0.49% ID/g for ^68^Ga-DOTA-NMN and 4.89 ± 0.53% ID/g of ^68^Ga-DOTA-NMN-AuNP). There are no significant accumulation in organs (*p* > 0.19) other than the kidneys (21.14 ± 1.97% ID/g for ^68^Ga-DOTA-NMN; 35.72 ± 4.83% ID/g for ^68^Ga-DOTA-NMN-AuNP; *p* = 0.041), liver, lungs and tumour (2.02 ± 0.02% ID/g of ^68^Ga-DOTA-NMN and 2.57 ± 0.26% ID/g ^68^Ga-DOTA-NMN-AuNP, *p* = 0.035), which indicates receptors mediated uptake as they express neurotensin receptors [20].

In the case of mice with prostate cancer (Figure 8b) there is a slower renal excretion after 30 min p.i. as 58.34 ± 9.86% ID/g for ^68^Ga-DOTA-NMN is found in the kidneys. After the ^68^Ga-DOTA-NMN-AuNP injection, the renal excretion of the tracer is improved, decreasing to 37.07 ± 8.11% ID/g but keeping similar values in the blood (5.69 ± 0.59% ID/g for ^68^Ga-DOTA-NMN-AuNP compared to 5.85 ± 0.83% ID/g for ^68^Ga-DOTA-NMN; *p* = 0.028). Furthermore, tumour uptake is doubled from 1.77 ± 0.5% ID/g for ^68^Ga-DOTA-NMN to 3.29 ± 0.08% ID/g for ^68^Ga-DOTA-NMN-AuNP with statistical significance (*p* = 0.09). Nanoparticles also double the uptake in the liver, small intestine and stomach.

## 3. Discussion

In this study, we successfully labelled with a positron emitter (^68^Ga) DOTA-neuromedin B, DOTA-neuromedin N, bombesin analogue DOTA-PEG(4)-BBN(7-14) and neurotensin DOTA-NT peptide. The labelling of the four peptides with ^68^Ga radioisotope was performed in semi-automated synthesis module, the synthesis process being optimised to obtain a labelling yield higher than 80% and radiochemical purity higher than 80% for ^68^Ga-DOTA-PEG(4)-BBN(7-14), and up to 100% for ^68^Ga-DOTA-NT and ^68^Ga-DOTA-NMN. The final compounds contain no unbound ^68^Ga and are stable for at least 4 h post final purification, being suitable for biological tests.

After the functionalization process of gold nanoparticles with the above-mentioned peptides we evaluated the in vitro uptake, retention, and specific binding of each tracer on human colon cancer cells HT-29 and DU-145 prostate cancer cells. Almost all tracers formed stable bonds with their specific receptors, except for ^68^Ga-DOTA-NMB for which the uptake and cell retention profile varies during the analysis. The highest retention value was 75%, measured for ^68^Ga-DOTA-PEG(4)-BBN(7-14)-AuNP on DU-145 prostate cancer cells.

Ex vivo biodistribution studies in immunocompromised mice inoculated with HT-29 and DU-145 tumour cells confirmed the results obtained in vitro, showing that cell retention was significantly improved after the incubation of cells with peptide-functionalized gold nanoparticles. Due to the ability of NHS-activated PEG coating of gold nanoparticles to react with the primary amines of peptides, an external ^68^Ga-labelled peptide layer is formed [21], and the signal detected in the tumour tissue is doubled in prostate tumours for ^68^Ga-DOTA-NMN-AuNP, as well as in colon tumours for ^68^Ga-DOTA-NMB-AuNP and ^68^Ga-DOTA-NT-AuNP. In the case of ^68^Ga-DOTA-PEG(4)-BBN(7-14)-AuNP on both colon and prostate tumours, the activity of ^68^Ga is three times higher than that obtained for ^68^Ga-DOTA-PEG(4)-BBN(7-14). Peptide functionalized gold nanoparticles can be delivered to tumours by both passive targeting mechanism through EPR effect; and by active targeting mechanism, as NT/NMN and BBN/NMN peptides recognise specific receptors on colon and prostate cells. Thereby, tumour neo-vascularisation can influence the uptake of nanoparticles into tumour cells [8]. A slight increase in ^68^Ga-DOTA-NMB-AuNP uptake was observed also in prostate tumours as well as ^68^Ga-DOTA-NMN-AuNP uptake in colon tumours, but further studies are needed to assess whether this slightly lower outcome (compared to the other peptides-AuNPs systems) was influenced by poorly vascularised or necrotic tumour tissues.

Previous studies for nanoparticle-based drug development highlighted the importance of reticuloendothelial system (RES), a network of cells and tissues responsible for avid clearance of unmodified NPs from the blood circulation within seconds or minutes [8,9,22]. Among the essential organs of RES, spleen, kidneys, liver, pancreas, lungs, bone marrow and lymph nodes play an important role in the pharmacokinetics of nanoparticle-based pharmaceuticals, and for this reason special attention has been paid to the amount of tracer absorbed in these tissues. In the case of ^68^Ga-DOTA-NMB (-AuNP) and of ^68^Ga-DOTA-NMN injected into mice with prostate cancer, although the clearance of tracers from the bloodstream is fast, the renal excretion is slower compared with the other peptides, with large amounts of tracers found at 30 min p.i. in the kidneys (approx. 60% ID/g). As neurotensin-like receptors are also found in the kidneys [20], the uptake of ^68^Ga-DOTA-NMN is most likely due to the interaction of neuromedin N with its receptors, which still needs to be studied to fully understand its nature.

Liver and biliary system represents the primary route for excretion of nanoparticles that do not undergo renal clearance, of which Kupffer cells are a potential site for toxicity as they express receptors for selective endocytosis of opsonized nanoparticles and rely on particle degradation for clearance [9]. Thus, nanoparticles that cannot be degraded by intracellular processes will remain within the cells and will therefore be retained. The role of polyethylene glycol (PEG 5 kDa) is to prevent the opsonin proteins from passively absorbing on the nanoparticles surface, and thus to prevent unwanted accumulations of NPs in the liver. However, slightly increased ^68^Ga-DOTA-PEG(4)-BBN(7-14)-AuNP activities were measured in mice with both colon and prostate cancer, and higher levels of ^68^Ga-DOTA-NMN-AuNP (doubled compared to ^68^Ga-DOTA-NMN) in mice inoculated with DU-145 prostate cancer cells.

Previous studies revealed that NPs size strongly influence the route by which they are excreted/eliminated from the system; nanoparticles with hydrodynamic diameter less than 6 nm are primary filtered by the kidneys [23], NPs <200 nm undergo liver excretion mediated by Kupffer cells, while NPs >200 nm by splenic macrophages excretion. We observed 3.19 ± 1.39% ID/g ^68^Ga-DOTA-NT-AuNP (with hydrodynamic diameter of 72.36 nm) in the spleen, for mice inoculated with colon cancer cells, which suggests receptor mediated uptake [24].

Several tracers have been developed to target and facilitate the molecular imaging of colon or prostate cancer, such as: ^68^Ga-NGR with over 2.7 ± 0.65% ID/g in colon tumour at 30 min p.i. [25], [^18^F]FDG with 2.5 ± 0.5% ID/g [^18^F]FDG in colon tumours [26], [^18^F]fluorocholine (FCH) with 7.9 ± 5% ID/g at 60 min p.i. in prostate tumour bearing mice [27]. Compared to these, the values obtained for tumour uptake using the four studied nanotracers are between 2.53 ± 0.26% ID/g for ^68^Ga-DOTA-NMN-AuNP to 9.82 ± 2.28% ID/g for ^68^Ga-DOTA-PEG(4)-BBN(7-14)-AuNP in colon tumours and 2.79 ± 0.4% ID/g for ^68^Ga-DOTA-NMB-AuNP to 13.01 ± 2.49% ID/g for ^68^Ga-DOTA-PEG(4)-BBN(7-14)-AuNP. Despite the promising findings, the advantages of bombesin, neuromedin B, neurotensin and neuromedin N-functionalized gold nanoparticles have only been demonstrated in vitro and ex vivo. In vivo μPET/CT imaging using these radiolabelled nanocarriers will be investigated in our future studies along with quantitative evaluation by ICP-MS to confirm that the peptides do not detach from the gold nanoparticles in physiological fluids.

## 4. Materials and Methods

### 4.1. Peptides Labelling

The peptides used in this work are commercially available and were purchased already coupled with DOTA chelator from Phoenix Europe GmbH (Manheim, Germany; DOTA-NT and DOTA-NMB), AnaSpec Inc. (Fremont, CA, USA; DOTA-PEG(4)-BBN(7-14)) and PolyPeptide (Strasbourg, France; DOTA-NMN), respectively. ^68^Ga used for labelling was obtained from a GMP-grade ^68^Ge/^68^Ga generator (ITG Isotope Technologies Garching GmbH, Munich, Germany) eluted with 0.05 M HCl prepared with suprapure HCl (Merck KGaA, Darmstadt, Germany). Labelling pH was adjusted with ammonium acetate (Carl Roth GmbH, Karlsruhe, Germany), used as 1 M solution. Separation and purification of labelled peptides were performed on Strata™-X solid phase extraction cartridges (Phenomenex Inc., Torrance, CA, USA). Ethanol (Chimreactiv, Bucharest, Romania) was used to recover the compound retained on cartridge. After evaporation of ethanol, the labelled compounds were dissolved in 1 mL saline solution (0.9% NaCl, B. Braun Melsungen AG, Melsungen, Germany), then finally filtered through a 0.22 μm pore size filter (Merck KGaA). Ultrapure water used for solutions preparation and for rinsing was freshly prepared in-house on a Millipore Milli-Q Direct 8 (Merck KGaA). The ^68^Ge/^68^Ga generator was coupled to a programmable synthesis module Galigand GAL-102 (Veenstra Instruments, Joure, The Netherlands). The whole preparation process was implemented and remotely controlled on Galigand GAL-102 labelling module. The peptides were previously dissolved in ultrapure water. Aliquots of 20 nmol of peptide per 50 μL solution were used for each synthesis, except DOTA-PEG(4)-BBN(7-14) for which 40 nmol per 50 μL solution were used. Prior of being loaded into reaction vial, the peptide must be protected by adding a certain amount of buffer (ammonium acetate 1 M). The amount of buffer necessary to adjust pH in the range of 3.8–4.3, suitable for the labelling reaction, was previously determined [28]. In order to be used for biomolecules labelling, ^68^Ga was eluted from the generator using 0.05 M HCl. At this pH value, the gallium is introduced into the process as gallium (III) chloride. Exactly 2 mL of gallium eluate, containing the highest amount of eluted activity, was added over the buffered peptide already loaded into the reaction vial. To shorten the overall preparation time, the labelling temperature was increased to 95 °C; at this value, the duration required for the labelling step is 5 min. The reaction mixture is then passed on the separation cartridge which retains the peptide, while the impurities are evacuated to waste. After rinsing on cartridge by passing 1 mL water with 5% ethanol content, the peptide was recovered with 1 mL of ethanol and sent to the evaporation vial, where the ethanol was evaporated to dryness under vacuum and heating (95 °C). The peptide was dissolved in 1 mL saline solution and transferred to the final product vial by passing through a 0.22 μm pore size filter for sterilization purpose.

### 4.2. Quality Control of ^68^Ga-Labelled Peptides

Activity was measured using the Atomlab™ 500 dose calibrator (Biodex Medical Systems Inc., Shirley, NY, USA). RCP and stability of the radiolabelled peptides were assessed by radio-HPLC performed onn a HPLC Shimadzu Prominence 20A system (Shimadzu, Kyoto, Japan) equipped with FlowStar LB 513 bismuth germanate γ detector (Berthold Technologies GmbH & Co.KG, Bad Wildbad, Germany). The stationary phase used was Nucleosil^®^ C18 column (5 μm, 250 mm × 4.6 mm—Merck KGaA) and the mobile phase consisted of water and acetonitrile, both acidified with 0.1% TFA. pH was evaluated with pH paper for 3.6–6.1 and 2.0–9.0 ranges (Macherey-Nagel, Duren, Germany). The activity loaded in the final vial was measured by dose calibrator and used for calculation of the specific activity and for yield evaluation. Due to the short half-life of ^68^Ga radioisotope, the value had to be decay-corrected for the moment of use, in order to keep a rigorous control over the activity added in biological tests. For accurate calculation of activity used in biological tests, the value of exact activity used was calculated by difference, measuring the activity of the syringe before and after adding the radiolabelled compound over the biological sample. In order to check the reproducibility of the preparation process, along with the main compound activity, the values of residual activity of the key components were measured and decay-corrected for the reference time. The preparation yields were then calculated based on these sets of values. Prior of using the final compound to biological tests, its pH was checked. A radio-HPLC method was used to assess the radiochemical purity of the final compounds, and the stability of the radiolabelled molecules. RCP was checked every hour, up to 4 h after the end of synthesis.

### 4.3. Nanoparticles Functionalization

**Figure 9:**
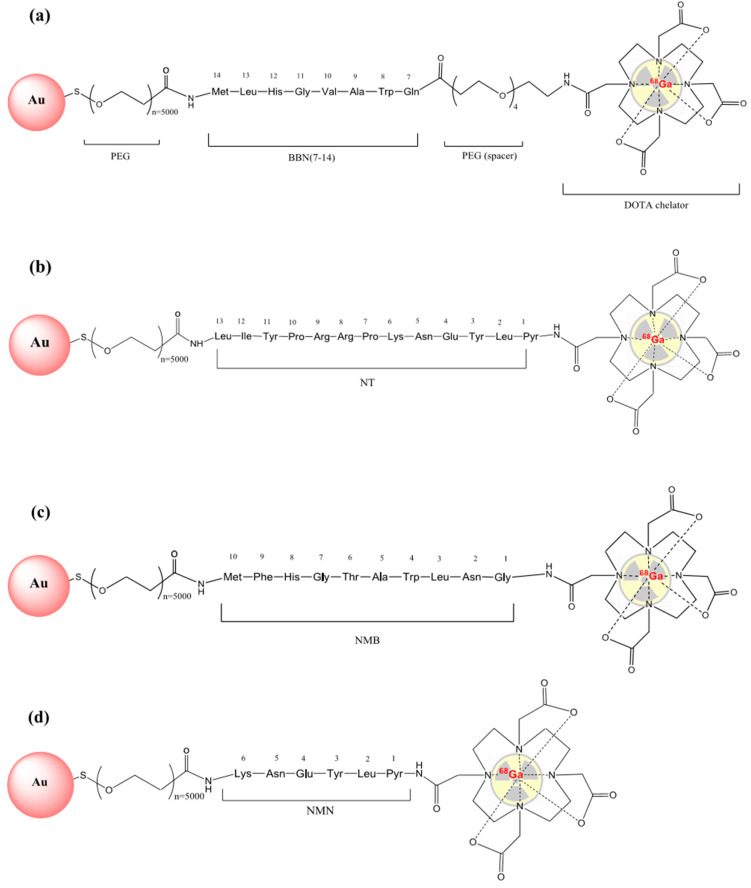
The structure of peptide-AuNP nanocarriers labelled with ^68^Ga: (**a**) ^68^Ga-DOTA-PEG(4)-BBN(7-14)-AuNP; (**b**) ^68^Ga-DOTA-NT-AuNP; (**c**) ^68^Ga-DOTA-NMB-AuNP; (**d**) ^68^Ga-DOTA-NMN-AuNP.

Ready-to-use kits containing pre-made mixtures for peptides functionalization on 40 nm NHS-activated gold nanoparticles conjugated with 5 kDa PEG-spacer were purchased from Cytodiagnostics (Burlington, ON, Canada). The functionalization process was conducted according to the protocol provided by the manufacturer [29]. The conjugation kit consists of NHS-activated gold NPs, reaction buffer, protein resuspension buffer and quencher solution. One vial with ~1 mg NHS-activated gold nanoparticles was mixed with 12 nmol of each ^68^Ga-labelled peptide (^68^Ga-DOTA-PEG(4)-BBN(7-14), ^68^Ga-DOTA-NT, ^68^Ga-DOTA-NMB, ^68^Ga-DOTA-NMN) and 60 µL of reaction buffer. The peptide-AuNP solution was then stirred at room temperature for 30 min. At the end of the functionalization process 10 µL of quencher solution was added to stop the reaction. The structures of the obtained nanocarriers are presented in Figure 9.

### 4.4. Physicochemical Characterisation

#### 4.4.1. DLS

The size distribution and zeta potential of cold peptide functionalized-gold nanoparticles were measured using a Nano ZS zetasizer system (Malvern Instruments, Malvern, UK). Measurement parameters were as follows: a laser wavelength of 633 nm (He-Ne), measurement angle of 90°, measurement temperature of 25 °C, and refractive index of medium *n* = 1.330.

#### 4.4.2. FTIR Spectra

FTIR spectroscopy was performed using KBr (IR grade) pellets containing the sample over the 5000–350 cm^−1^ wavenumber range with a model 6100.4.5 FTIR spectrophotometer (Jasco, Easton, MD, USA).

### 4.5. Cell Culture and Petri Dishes Preparation

Biological investigations were performed on human normal cell lines (proliferating human CRL monocytes) and tumour cell lines (human colon carcinoma HT-29 cells and human prostate carcinoma DU-145 cells), purchased from the American Cell and Tissue Collection (ATCC, Manassas, VA, USA). Cells were maintained in culture according to the protocol provided by the depositor. Human CRL9855 cells were cultivated in IMDM culture medium (ThermoFisher Scientific, Wiltham, MA, USA) supplemented with 10% fetal bovine serum (FBS, Merck), HT solution (100x: 10 mM sodium hypoxanthine and 1.6 mM thymidine, Thermo Fisher Scientific) and 0.05 mM 2-mercaptoethanol (Sigma-Aldrich, Darmstadt, Germany). Adherent HT-29 and DU-145 cells were cultivated in DMEM-F12 culture medium (ThermoFisher Scientific) supplemented with 10% fetal bovine serum (FBS, Merck) and antibiotic-antimycotic solution (Sigma-Aldrich, Darmstadt, Germany). Passage of cells was done every 2–5 days for maintaining an appropriate density of cells in culture. For adherent cells split a 0.25%/0.02% trypsine/EDTA solution (Biochrom Ltd., Cambridge, UK) was used. Cells at passage 6–9 and were counted before seeding for experimentation by optical microscopy using a Burker-Turk counting chamber, and cell viability was assessed by the trypan blue exclusion test. Only cell suspensions with viability above 95% were used in experiments. 24 h prior to a binding experiment with LigandTracer Yellow^®^ (Ridgeview Instruments AB, Uppsala, Sweden), Petri dishes (Nunc™ 150350, ThermoFisher Scientific) were prepared with approximately 4 × 10^5^ cells. The cells were seeded on a well-defined area of a tilted cell culture dish and incubated with 2 mL DMEM medium for 12 h. Before the experiment, the Petri dish is verified under microscope to check for cells migration outside the area of interest.

### 4.6. Cytotoxicity Investigation

The effect of AuNP NPs loaded with neuropeptides (DOTA-PEG(4)-BBN(7-14), DOTA-NMB, DOTA-NMN, DOTA-NT) on normal proliferating cells (human CRL9855 cells) or human tumour cells (human colon carcinoma HT-29 cells and human prostate carcinoma DU-145 cells) was investigated using the MTS reduction test (CellTiter 96^®^ Aqueous One Solution Cell Proliferation Assay, Promega, Madison, WY, USA), as indirect measure of the number of metabolically active cells in culture. Briefly, cells were cultivated in 96-well plates (3 × 10^4^ CRL9855 cells/mL, 10 × 10^4^ HT-29 cells/mL and 5 × 10^4^ DU-145 cells/mL) in a total volume of 100 μL. Adherent cells were left for 24 h to adhere to the culture well. Thereafter, NPs and peptides were added at various concentrations and cells were cultivated for 48 h. Non-treated cells were considered as control (CTRL). For background assessment culture medium, NPs and peptides were cultivated in absence of cells, considering that NPs might interfere with the spectrophotometric measurement. All samples were cultivated in triplicate. At the end of the incubation time, 20 μL of MTS reagent were added and samples were cultivated for another 1–3 h until the reaction color was developed. The OD at 490 nm was measured in each sample using a Tecan Sunrise ELISA reader with Magellan universal reader control and data analysis software (Tecan Group Ltd., Mannedorf, Switzerland). Final OD in cellular samples was calculated by subtracting the OD of the corresponding background samples. Results were presented as mean ± standard error of the mean (SEM) for triplicate samples.

### 4.7. In Vitro Binding Studies

#### 4.7.1. Uptake and Retention Assay

The interaction between ^68^Ga-labelled tracers and living cells expressing a target receptor was measured in real-time using rotating radioimmunoassay (RIA) method implemented on the automatic LigandTracer^®^ Yellow system [30], by calculating at each rotation the difference between the signals detected from 6 points in the background area of the Petri dish and 6 points from the area of interest. The binding curve is obtained after a two-phase process consisting of an incubation phase and a wash-out phase. In the first phase, a background point is acquired after which 2–3 nmol of peptide-containing tracer is incubated over the cells for a time equal to that required to reach the equilibrium, which is recognized on the graph by a plateau trend. After this phase, the radioactive medium was carefully extracted from the dish, ensuring that the liquid did not exceed the defined area, and the cells were washed twice with 1 mL of DMEM medium. At the end, 2 mL of fresh medium is added over the cells to ensure optimal survival conditions during the next phase. In the wash-out phase, the retention of the tracer to cancer cells is evaluated. By removing the radioactive medium, the uptake process is stopped, the signal detected coming from ^68^Ga radioisotope coupled to the receptor-bound peptides. This part is measured for a time long enough to permit to assess the stability of the interaction. Since kinetic parameters are dependent only on the amount of incubated peptide and the incubation time, signal values were normalized to 0% at baseline level and 100% at the end of the incubation phase, respectively, to enable easy visual comparison of interaction profiles between different peptides.

#### 4.7.2. Specific Binding Assay

Specific uptake of ^68^Ga-labelled peptides at their receptors was assessed using non-peptide antagonists capable of blocking receptors prior to incubation of peptides. The antagonists selected to block the receptors were chosen based on their affinity for the three receptors of each peptide. For accuracy, a combination of several antagonists was used to block all receptors before incubating the tracers. Thus, ML 18 and PD 176252 antagonists were used to prevent ^68^Ga-DOTA-BBN(7-14) and ^68^Ga-DOTA-Neuromedin N from binding to bombesin receptors (BBRS1, BBRS2 and BBRS3) and SR 142948, SR 48692 and NTRC 824 to block the neuromedin N peptide binding to neurotensin receptors (NTRS1, NTRS2 and NTRS3). ML-18 is a non-peptide bombesin receptor subtype 3 (BBRS3) antagonist for which has been recently demonstrated its ability to inhibit the growth of lung cancer [31]. PD 176252 is a non-peptide gastrin-releasing peptide receptor (GRP-R) antagonist also known as bombesin receptor subtype 2 (BBRS2), and neuromedin B receptor (NMB-R) antagonist also known as BBRS1 [32]. NTRC 824 is selective for neurotensin receptor subtype 2 (NTRS2) over NTRS1, by contrast, SR 48692 is selective for NTRS1 over NTRS2 but not NTRS3. For this reason, a third neurotensin receptor antagonist was used, namely SR 14294.

Predefined amounts of each antagonist presented in Table 3 were incubated over the cells for 1–1.5 h followed by removal of the medium, washing the cells with DMEM medium and incubation of the radiolabeled peptides according to the protocol described above.

### 4.8. Ex Vivo Biodistribution

All experiments were conducted according to the guidelines of EU Directive 2010/63 approved by The Romanian National Sanitary Veterinary and Food Safety Authority (NSVFSA, approval no. 442/25.02.2019). Athymic nude male mice (NU/J, 8–10 weeks old) were obtained from The Jackson Laboratory (Bar Harbor, ME, USA). Animals were housed in ventilated cages with a 12-h light-dark cycle and receive food and water ad libitum. Colon/prostate tumours were induced 7–10 days prior the experiments by a subcutaneous injection of 1 × 10^6^ HT-29/DU-145 cancer cells. All injected animals were monitored daily until an ideal size of tumour for biodistribution was reached (60–100 mm^3^). 3.81 ± 0.51 MBq of ^68^Ga-labelled tracers were retro-orbital injected into the ophthalmic venous sinus after intraperitoneal anesthesia with 0.1 mL ketamine/xylazine/acepromazine cocktail: 7.5 mg/mL ketamine (Narkamon Bio, Bioveta, Cluj-Napoca, Romania), 1.5 mg/mL xylazine (Narcoxyl 2, MSD Animal Health, Madison, NJ, USA) and 0.25 mg/mL acepromazine (Sedam, SC Pasteur Filiala Filipesti SRL, Filipestii de Padure, Romania). All animals were sacrificed at 30 min p.i. and the organs were weighted and measured in a dose calibrator (Biodex Medical Systems Inc., Shirley, NY, USA). The biodistribution was calculated as percent of injected dose per gram of organ (% ID/g) ± standard deviation (SD, *n* = 4 for each injected tracer).

### 4.9. Statistical Analysis

All the data were expressed as means ± standard deviation (*n* = 4). Comparison of results among the groups (% ID/g vs. tracers, % ID/g vs. tracers and organs, % ID/g vs. tracers and tumoral cells), was carried out by one-way analysis of variance (ANOVA) and Tukey post-hoc analysis using SPSS v26 (IBM, Armonk, NY, USA) The results were considered significant for a *p*-value less than 0.05.

## 5. Conclusions

In conclusion, we synthetized and evaluate both in vitro and ex vivo in tumour-bearing immunocompromised mice four different gold nanocarriers for ^68^Ga-labelled bombesin and neurotensin analogues that exhibited high stability in physiological conditions, minimum accumulation in non-targeted organs and fast distribution in colon and prostate tumour tissue. Promising results were obtained for ^68^Ga-DOTA-PEG(4)-BBN(7-14)-AuNP and ^68^Ga-DOTA-NT-AuNP, which we propose for future multimodal imaging PET/CT studies.

## Figures and Tables

**Table 1:** The overall preparation yield of radiolabelled peptides.

Peptide	Preparation Yield (%)
^68^Ga-DOTA-NT	82.12 ± 3.43
^68^Ga-DOTA-NMN	81.14 ± 3.35
^68^Ga-DOTA-PEG(4)-BBN(7-14)	80.70 ± 3.10
^68^Ga-DOTA-NMB	80.60 ± 0.46

**Table 2:** Nanocarriers physical properties as measured by DLS.

Nanostructure	Hydrodynamic Diameter	Zeta Potential
DOTA-PEG(4)-BBN(7-14)-AuNP	71.05 nm	−44.7 mV
DOTA-NMB-AuNP	72.18 nm	−47.3 mV
DOTA-NT-AuNP	72.36 nm	−44.4 mV
DOTA-NMN-AuNP	73.29 nm	−49.6 mV

**Table 3:** The amounts of antagonists used to evaluate specific binding.

Antagonist	Target Receptors	K_d_	Incubation Quantity	Incubation Time
PD 176252	BBRS1, BBRS2	1 nM	1 nM	1 h
ML 18	BBRS3	-	1 nM	1 h
SR 48692	NTRS1, NTRS2	15 nM	30 nM	1.5 h
NTRC 824	NTRS1, NTRS2	38 nM	70 nM	1.5 h
SR 14294	NTRS1, NTRS2, NTRS3	10 nM	10 nM	1.5 h

## Appendix B

**Table A1:** Ex vivo biodistribution data on NU/J mice inoculated with HT-29 colon cancer cells.

Tissue	^68^Ga-DOTA-PEG(4)-BBN(7-14)	^68^Ga-DOTA-PEG(4)-BBN(7-14)-*AuNP*	^68^Ga-DOTA-NMB	^68^Ga-DOTA-NMB-*AuNP*	^68^Ga-DOTA-NT	^68^Ga-DOTA-NT-*AuNP*	^68^Ga-DOTA-NMN	^68^Ga-DOTA-NMN-*AuNP*
Blood	6.42 ± 0.27	8.24 ± 0.86	4.72 ± 0.43	3.64 ± 0.32	5.79 ± 0.39	8.92 ± 0.17	4.59 ± 0.49	4.89 ± 0.53
Liver	1.92 ± 0.07	2.81 ± 0.57	1.82 ± 0.33	1.70 ± 0.35	1.55 ± 0.29	1.27 ± 0.83	1.23 ± 0.20	1.51 ± 0.80
Kidneys	18.74 ± 0.67	14.86 ± 1.32	36.23 ± 4.83	19.17 ± 5.77	24.00 ± 4.37	14.13 ± 1.31	21.14 ± 1.97	35.72 ± 4.83
Pancreas	2.13 ± 0.07	7.18 ± 1.63	0	1.99 ± 0.02	0.90 ± 1.05	8.34 ± 4.24	0	0.69 ± 0.11
Spleen	1.31 ± 0.22	1.69 ± 0.26	0.05 ± 0.01	0.56 ± 0.12	1.12 ± 0.52	3.19 ± 1.39	0.35 ± 0.02	0
Small intestine	1.70 ± 0.44	0.93 ± 0.25	0.43 ± 0.30	0.99 ± 0.06	1.30 ± 0.86	0.76 ± 0.25	0.43 ± 0.19	0.75 ± 0.33
Large intestine	2.63 ± 0.34	3.31 ± 0.56	0.38 ± 0.14	1.84 ± 0.51	1.48 ± 0.29	3.37 ± 1.66	0.84 ± 0.29	0.75 ± 0.24
Stomach	2.10 ± 0.50	0.98 ± 0.58	0.86 ± 0.24	0.85 ± 0.65	1.67 ± 0.46	0.49 ± 0.23	0.71 ± 0.17	0.94 ± 0.04
Lungs	4.17 ± 1.37	2.65 ± 0.11	1.85 ± 0.06	2.27 ± 0.04	2.63 ± 0.63	1.88 ± 0.11	2.37 ± 0.77	1.32 ± 0.10
Muscle	0.79 ± 0.21	1.03 ± 0.41	0.34 ± 0.03	0.53 ± 0.06	0.87 ± 0.27	0.93 ± 0.74	0.48 ± 0.32	0.33 ± 0.16
Tumour	3.31 ± 0.76	9.82 ± 2.28	1.73 ± 0.08	4.75 ± 0.32	4.06 ± 0.27	9.04 ± 2.29	2.02 ± 0.02	2.57 ± 0.26
Brain	0.49 ± 0.02	0.15 ± 0.03	0	0	0.66 ± 0.12	0.35 ± 0.13	0.04 ± 0.01	0.05 ± 0.01
Bone	0.75 ± 0.06	2.21 ± 0.51	0.10 ± 0.07	0	0.84 ± 0.22	2.71 ± 0.80	0.16 ± 0.02	0.13 ± 0.02
Lymph Node	1.10 ± 0.45	2.03 ± 1.23	0.49 ± 0.17	0	1.47 ± 0.27	0	0.39 ± 0.05	0
Heart	2.24 ± 0.12	1.75 ± 0.07	0.91 ± 0.05	0.51 ± 0.12	1.85 ± 0.24	1.56 ± 0.36	1.00 ± 0.42	0.32 ± 0.09

**Table A2:** Ex vivo biodistribution data on NU/J mice inoculated with DU-145 prostate cancer cells.

Tissue	^68^Ga-DOTA-PEG(4)-BBN(7-14)	^68^Ga-DOTA-PEG(4)-BBN(7-14)-*AuNP*	^68^Ga-DOTA-NMB	^68^Ga-DOTA-NMB-*AuNP*	^68^Ga-DOTA-NT	^68^Ga-DOTA-NT-*AuNP*	^68^Ga-DOTA-NMN	^68^Ga-DOTA-NMN-*AuNP*
Blood	6.48 ± 1.53	8.34 ± 0.05	7.00 ± 0.24	5.06 ± 1.51	7.77 ± 0.23	8.97 ± 0.79	5.85 ± 0.93	5.69 ± 0.59
Liver	2.22 ± 0.70	3.23 ± 0.39	1.53 ± 0.21	1.33 ± 0.51	1.82 ± 0.34	2.19 ± 0.15	1.10 ± 0.07	2.18 ± 0.76
Kidneys	17.96 ± 3.78	25.00 ± 6.87	60.28 ± 13.01	67.51 ± 4.16	24.4 ± 0.18	33.26 ± 7.61	58.34 ± 9.86	37.07 ± 8.11
Pancreas	2.51 ± 0.72	1.89 ± 0.63	1.36 ± 0.07	0	1.15 ± 0.63	0	0.58 ± 0.02	0
Spleen	0.57 ± 0.02	1.22 ± 0.34	0.73 ± 0.29	0	1.60 ± 0.53	1.92 ± 0.11	0	1.08 ± 0.36
Small intestine	1.13 ± 0.69	0.67 ± 0.27	1.43 ± 0.08	1.40 ± 0.14	1.92 ± 0.32	1.24 ± 0.17	0.26 ± 0.01	0.89 ± 0.05
Large intestine	1.30 ± 0.25	1.59 ± 1.37	0.64 ± 0.91	1.41 ± 0.10	2.14 ± 0.12	2.59 ± 0.44	0.13 ± 0.27	0.17 ± 0
Stomach	1.87 ± 0.03	1.61 ± 0.16	1.05 ± 0.25	1.42 ± 0.02	1.80 ± 1.19	3.96 ± 0.61	0.40 ± 0.33	0.96 ± 0.12
Lungs	4.59 ± 2.25	3.24 ± 0.91	4.08 ± 1.18	2.14 ± 0.79	4.51 ± 0.57	6.67 ± 0.82	2.37 ± 0.50	2.52 ± 0.24
Muscle	0.94 ± 0.14	0.63 ± 0.33	0.84 ± 0.07	0.92 ± 0.16	1.02 ± 0.20	1.78 ± 0.13	0.57 ± 0.22	0.71 ± 0.23
Tumour	3.78 ± 0.27	13.01 ± 2.49	2.54 ± 0.42	2.79 ± 0.44	3.78 ± 0.05	11.7 ± 0.59	1.77 ± 0.50	3.29 ± 0.08
Brain	0.21 ± 0.04	0.57 ± 0.09	0	0.11 ± 0.03	0	0.64 ± 0.19	0	0.03 ± 0.01
Bone	0	0	0	0.24 ± 0.02	1.03 ± 0.58	0.48 ± 0.35	0	0.13 ± 0.02
Lymph Node	0	0	1.25 ± 0.69	0	2.23 ± 0.49	3.04 ± 1.13	0	0.27 ± 0.03
Heart	1.45 ± 0.13	1.04 ± 0.48	2.22 ± 0.12	0.93 ± 0.19	3.12 ± 0.64	3.13 ± 0.24	0.99 ± 0.26	0.17 ± 0.09
